# Cas9-chromatin binding information enables more accurate CRISPR off-target prediction

**DOI:** 10.1093/nar/gkv575

**Published:** 2015-10-10

**Authors:** Ritambhara Singh, Cem Kuscu, Aaron Quinlan, Yanjun Qi, Mazhar Adli

**Affiliations:** 1 University of Virginia, School of Medicine, Department of Biochemistry and Molecular Genetics, 1340 Jefferson Park Ave, Jordan Hall, Room: 6240, Charlottesville, VA 22903, USA; 2 University of Virginia, Department of Computer Science, Charlottesville VA 22903, USA; 3 University of Virginia, Center for Public Health Genomics, Charlottesville VA 22903, USA; 4 University of Virginia, Department of Public Health Sciences, Charlottesville VA 22903, USA

## Abstract

The CRISPR system has become a powerful biological tool with a wide range of applications. However, improving targeting specificity and accurately predicting potential off-targets remains a significant goal. Here, we introduce a web-based **CR**ISPR/Cas9 **O**ff-target **P**rediction and **I**dentification **T**ool (CROP-IT) that performs improved off-target binding and cleavage site predictions. Unlike existing prediction programs that solely use DNA sequence information; CROP-IT integrates whole genome level biological information from existing Cas9 binding and cleavage data sets. Utilizing whole-genome chromatin state information from 125 human cell types further enhances its computational prediction power. Comparative analyses on experimentally validated datasets show that CROP-IT outperforms existing computational algorithms in predicting both Cas9 binding as well as cleavage sites. With a user-friendly web-interface, CROP-IT outputs scored and ranked list of potential off-targets that enables improved guide RNA design and more accurate prediction of Cas9 binding or cleavage sites.

## INTRODUCTION

The CRISPR/Cas9 system is a bacterial immune response system that has been adapted as an RNA-guided genome-editing tool (1–3). Due to its simple design and high efficiency, application areas for this exciting technology are ever expanding. The wild-type catalytically active Cas9 has being used in a wide range of organisms for targeted gene editing or for whole-genome genetic knockout screenings (4–8). On the other hand, the catalytically inactive version of Cas9 (dCas9) has even broader application areas such as gene regulation (4,9–11), screenings of genome-scale gene activation or repression (12), chromatin imaging (13) and chromatin (14) and RNA pull down (15) purposes.

In the Type II CRISPR system of *Streptococcus pyogenes*, the most widely utilized CRISPR system, targeting specificity is governed by 20-nt sequence of single guide RNA (sgRNA) and a Protospacer adjacent motif (PAM) sequence of NGG in the genomic sequence. Since the early studies demonstrated CRISPR as RNA guided gene-editing tool, multiple studies have assessed Cas9 targeting specificity (16–20) and concluded that CRISPR/Cas9 may cleave certain off-target site by tolerating limited mismatches in the PAM distal part of guiding RNA. Recently, others and we mapped *in* *vivo* binding activity of catalytically inactive Cas9 across the human and mouse genomes using ChIP-Seq technology and assessed WT Cas9 mediated DNA double strand breaks at the off-target binding sites (21–23). Furthermore, additional genome-wide methods have been proposed to monitor Cas9 mediated off-target cleavage sites (24–26). The emerging conclusion from all these whole genome off target Cas9 binding and cleavage studies suggested that Cas9-DNA binding requirements are different than Cas9 mediated DNA double strand breaks.

Importantly, CRISPR system is now adapted for multiple purposes. While RNA guided DNA double stand break activity of WT Cas9 is being heavily used for genome editing, DNA binding activity of catalytically inactive Cas9 is adapted for wide range of completely different purposes including gene regulation and chromatin pull down and imaging. It is expected that this flexible tool will be further repurposed for novel applications. For existing as well as future CRISPR applications, it is essential to maximize the targeting specificity and monitor the potential off-target Cas9 binding and cleavage sites in the genome. To this end, multiple off-target prediction algorithms and tools have been published for CRISPR system (16,27–34). Despite minor differences in their performance and respective output, these tools are based on pure DNA sequence similarity information only. Most tools allow a limited number of mismatches when exploring potential off-target sites (16,31–33) and yield a limited number of computationally predicted off-target sites (16,28,31). Moreover, some tools report potential off-target candidates without scoring or ranking the predictions (27,29). We previously noted that when solely DNA sequence information is used, only a small fraction of experimentally validated Cas9 bindings sites were predicted (21). We therefore explored novel ways to achieve better computational off-target prediction. In the light of recent whole genome Cas9 mapping studies, we developed CROP-IT tool that integrates knowledge from experimentally identified Cas9 binding sites, cleavage sites as well as chromatin state information. CROP-IT scores each predicted Cas9 site and outputs a user-defined list of top number of sites. The CROP-IT algorithm is based on a computational model where each position of the guiding RNA sequence is differentially weighted based on experimental Cas9 binding and cleavage site information from multiple independent sources (21–24). Furthermore, it incorporates chromatin state information for the human genome by analyzing accessible chromatin regions from 125 human cell types (35). By integrating observed information from Cas9 DNA binding, CROP-IT performs significantly better than existing computational prediction tools. CROP-IT provides a user-friendly web site where users can design optimal sgRNA guiding sequences and can search for potential off-target binding or cleavage sites.

## MATERIALS AND METHODS

### CROP-IT algorithm

Our tool takes the target sequence from the user as input. First, it aligns the target 20 bp sequence to the reference genome selected by the user, using PATMAN (36). It allows up to nine mismatches (binding) and six mismatches (cleavage) in the resulting alignment candidates. We chose these numbers because based on ChIP-Seq experiments, Cas9 binding allows as many as 9–10 mismatches at the off-target binding sites. On the other hand, whole genome Cas9 cleavage studies such as GUIDE-Seq experiments demonstrate that the system allows lower number (6-7) of mismatches. The list of ∼3 000 000 alignments in average from PATMAN is provided as input into our program that processes and scores the sequences according to the algorithm described below:

- Our program first performs a filtering step whereby solely those sequences ending with ‘NGG’ and/or ‘NNG’ are selected. Notably, the PAM sequence for CRISPR system was initially proposed to be GGG. However, recent studies show that NNG may also work as a PAM sequence. The unbiased off-target binding sites analysis of ChIP-Seq data suggest that the system prefers GGG over NGG, and NGG over NNG (21).
- According to the selected PAM option, the sequences are binned into separate files for each respective PAM output and hence, the scoring of sequences takes place for each file.
- Next, in order to score alignment candidates, the first 20 bp (sequences without PAM) are divided into three segments of 5, 5 and 10 base pairs each respectively. Different mismatch/match scores are applied for each of these three segments.
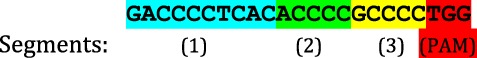
- Each nucleotide in the 20 bp region is compared to the target sequence. In the event of a match, the program assigns a score *s_i_* (where *i* = 1, 2,3) based on the segment it belongs to and in the event of a mismatch, it looks for a consecutive mismatch next to it. A penalty *–s_i_* is assigned when there are two consecutive mismatches in the 20 bp region, whereas penalty $(-s_i/2)$ is assigned for a single mismatch. The difference in penalty assignment is based on our observation of the experimental data, where Cas9 binding intensity was higher for sequences with small number of consecutive mismatches as compared to those with large number of such mismatches. This indicates that the former types of sequences are more likely to be off-target sites and therefore, are ranked higher in our predicted list. Thus,
  assigned score,
  \begin{equation*} S = \sum \limits _{i = 1}^3 [(n \times s_i ) +(m \times (-s_i)+k \times (-s_i/2))] \end{equation*}
  where, *n* = number of matches, *m* = number of consecutive mismatches and *k* = number of single mismatches.
- The values of *s* depend on the segment to which the nucleotide, being compared, belongs. Therefore, the first segment, numbered 1, is assigned score *s_1_* and penalty -*s_1_*. Similarly, the second and the third segments are assigned scores *s_2_* and *s_3_*; and penalties *-s_2_* and *-s_3_* respectively. The values of scores of all three segments were decided by performing non-parametric optimization, which has been described in subsequent section.
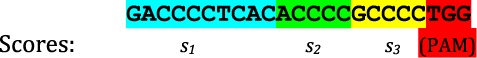
- For consecutive mismatches belonging to separate segments, the program takes the mean of the penalties for the two segments.
- Once the scoring of every sequence is complete, the algorithm generates output consisting of information describing the coordinates, sequences (with matches represented by capital letters), number of mismatches and assigned scores of each off-target site.

By allowing up to nine and six mismatches, our algorithm is capable of exploring a much larger substring space leading to better sensitivity.

### Weight optimization

The final string-match score is calculated as a weighted sum of matching and mismatching from all three segments,

assigned score,

\begin{equation*} S = \sum \limits _{i = 1}^3 [(n \times s_i ) +(m \times (-s_i)+k \times (-s_i/2))] \end{equation*}

where, *n* = number of matches, *m* = number of consecutive mismatches and *k* = number of single mismatches.

With the help of ChIP-Seq data collected from previous studies (21), we were able to learn the best values for weight parameters [*s_i_, i* = 1,2,3] through performing a grid-search based optimization. Grid search strategy is a technique to uniformly sample all possible values in order to decide the optimal weights. For learning the weights for our basic algorithm, we had to perform grid search on three parameters: *s_1_, s_2_* and *s_3_*. Initial starting values were selected randomly (min = 0 and max = 20). Next, the max values were extended with uniform spacing based on the performance of the algorithm on the sgRNA #4 dataset. For performance evaluation, we calculated the overlap of top 1000 predicted sites with actual ChIP-seq off-target sites. Max values were extended until the number of correctly predicted sites was reduced by 10 from the best performing number. This search problem is multivariate in nature, as we are tuning for three parameters and thus for *t* number of sampling values, our search space is of size *t^3^*. Since this space is feasible to explore, we tried weight values from total enumeration of all possible combinations of *s_1_, s_2_* and *s_3_*. This grid search gave the best performance for values *s_1_* = 5, *s_2_* = 70, *s_3_* = 50.


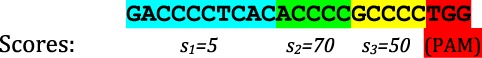


Finally, the performance of our algorithm with the best weight values was evaluated using independent test data obtained from study on mouse genome (Nanog and Phc1 ChIP-Seq data) by (22), thus reducing the likelihood of bias in our results.

### Incorporation of DNase I-Seq chromatin state data

Based on our observations after overlapping off-target sites with 125 cell type DNase I HS data, we decided to incorporate this information in our scoring system for human genome (hg19). In order to accomplish this, we used the trend line equation in graph in Figure 3A to differentially weigh genomic regions according to their frequency of having ‘open’ chromatin state in different cell types as measured by DNase I Sequencing. This relationship is captured by the additional score, which is summarized as follows.

Additional Score,

\begin{equation*} S_d = [0.0113 \times X \times d] \end{equation*}

where, *X* = number of cell types with that DNase I site common and *d* = weight assigned to a sequence if it overlaps with DNase I site and 0.0113 constant comes from the equation of the trend line in Figure 3A. Thus, after the initial scoring of potential off-target sequences by our algorithm, if the selected genome is human, we input the results into second round of scoring. Here, the additional score *S_d_* is added to the existing score, based on the site overlap with 125 cell type DNase I HS data. Therefore, our equation for human genome off-target scoring becomes:

Final Score,

\begin{equation*} \begin{array}{*{20}l} S_f = S + S_d \\ = \sum \limits _{i = 1}^3 \left [(n \times s_i ) +(m \times (-s_i)+k \times (-s_i/2))  \right ]+[0.0113 \times X \times d] \end{array} \end{equation*}

where, *n* = number of matches, *m* = number of consecutive mismatches, *k* = number of single mismatches, *X* = number of cell types common overlapping DNase I site and *d* = weight assigned to a sequence if it overlaps with DNase I site. The value of weight *d =* 20 was finalized after performing another round of grid search based non-parametric optimization on existing ChIP-Seq data for sgRNA#4 (described in previous section). CROP-IT now outputs an updated ranked list of potential off-targets based on incorporated information from DNase I data. For cleavage site prediction, we follow the procedure described above, beginning with allowing six mismatches in the 20 bp target sequence. While training on the cleavage site data, the weights obtained were as follows: *s*_1_ = 20, *s*_2_ = 60, *s*_3_ = 50 and *d* = 10. The training was performed using HEK293 Site 4 cleavage sites from (24) and the performance evaluation was done on VEGFA Site1, 2 and 3 respectively from the same study.

### Comparative analysis with other tools

We analyzed performance of CROP-IT with other state-of-the-art computational off-target prediction tools. These can broadly be categorized into two groups of tools; (A) that score off-targets (16,28,30,31,34) (B) do not score off-targets (26). For group (A), if the number of predicted off-targets were very large (>300 000), we took the top *n* (*n* = 500 to 10 000) ranked sites generated by the tool and CROP-IT and intersected the coordinates of predicted sites with those of ChIP-Seq data for the selected sgRNAs. We used BEDTOOLS (37) *intersect* (−*f* = 1) command for this purpose. For group (A) tools, where the number of predicted sites was limited, we selected all the sites (*N*) from the respective tool and chose the same number of top *N* sites of CROP-IT output list and performed the intersection. For group (B), we selected all predicted sites for CROP-IT (∼100 000) and all the sites of the other tool for evaluation. The results are reported as number of selected predicted off-target sites and reported binding or cleavage-sites.

## RESULTS

**Figure 1.**
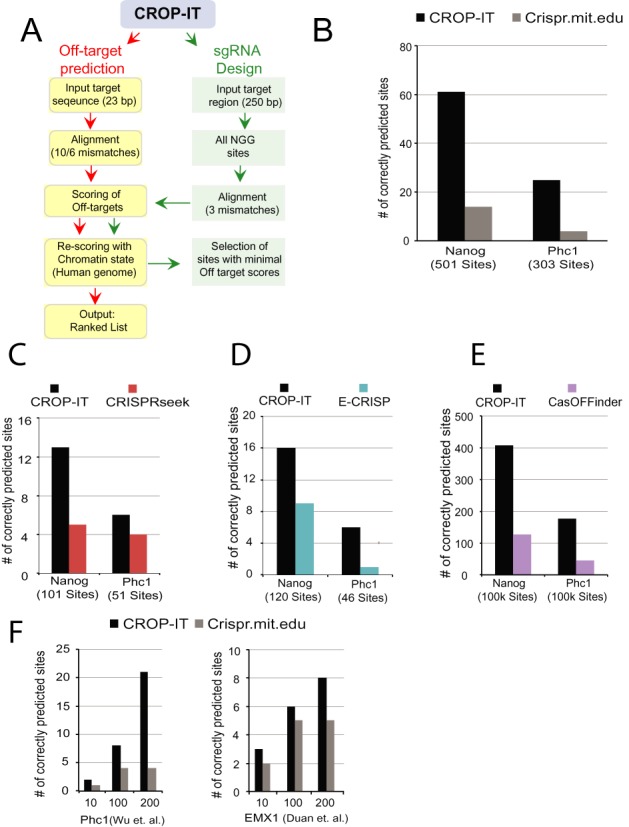
(**A**) Schematics of CROP-IT algorithm. Comparative analysis of prediction performance of CROP-IT (**B**) Crispr.mit.edu (16); (**C**) CRISPRSeek (28); (**D**) E-CRISP (31) and (**E**) CasOFFinder (29) for two different sgRNAs: *Nanog-sgRNA* and *PhC1-sgRNA*. Y-axis indicates the overlap between the maximum number of computationally predicted Cas9 binding sites for each tool and ChIP-Seq identified sites. The number of sites mentioned under the X-axis is top *N* sites for CROP-IT to match the number of predicted sites output by the other tool. For CasOFFinder, top 100 000 predicted sites (out of ∼6 000 000) were compared with those of CROP-IT (**F**) Comparison of CROP-IT and Crispr.mit.edu based on overlapping Cas9 binding sites with top 10, 100 and 200 predicted sites for Phc1 and EMX1 sgRNAs.

Several experimental methodologies that have been designed to understand targeting specificity of CRISPR/Cas9 system highlighted that the PAM-proximal portion of the guiding RNA is more critical for Cas9 targeting (16,19,21,22). In line with these studies, recent whole genome ChIP-Seq maps of catalytically inactive Cas9 binding sites (21,22) and independent whole genome Cas9 mediated cleavage detection assays such as HTGTS (25), Di-genome-Seq (26), GUIDE-Seq (24) and IDLV capture (38) tools have shown that the CRISPR system allows several mismatches in the PAM-distal part of the guiding sequence. These results also highlight the importance of monitoring both off-target binding as well as off-target cleavage activity of CRISPR system. Because RNA guided DNA binding activity of catalytically inactive Cas9 has wide range of applications independent of genome editing activity of catalytically active WT Cas9, CROP-IT has been devised to incorporate these experimentally validated features in its prediction algorithm. As summarized in Figure 1A, CROP-IT starts searching for potential off-target sites by allowing up to nine mismatches for binding sites and six mismatches for cleavage sites, in the 20-nt portion of the guiding sequence. The identified sites with NGG PAM sequence are scored based on a weighted computational simulation from non-parametric scoring algorithm using whole genome Cas9 ChIP-Seq binding data and GUIDE-Seq cleavage data sets from three independent sources (21,22,24). This novel string-matching method combines the matching and mismatching events from local segments into a weighted score, where the weights are learned through a grid-search based optimization strategy. Notably, we obtained optimum results when the guiding sequence of sgRNA is divided in to three parts: the PAM distal 10 bp seed sequence, the 5 bp middle sequence and the 5 bp PAM proximal sequence. Thus, by differentially weighting matches and mismatches in these three regions of sgRNA guiding sequence, CROP-IT scores and then ranks the identified off-target binding and cleavage sites.

### CROP-IT outperforms existing tools

**Figure 2.**
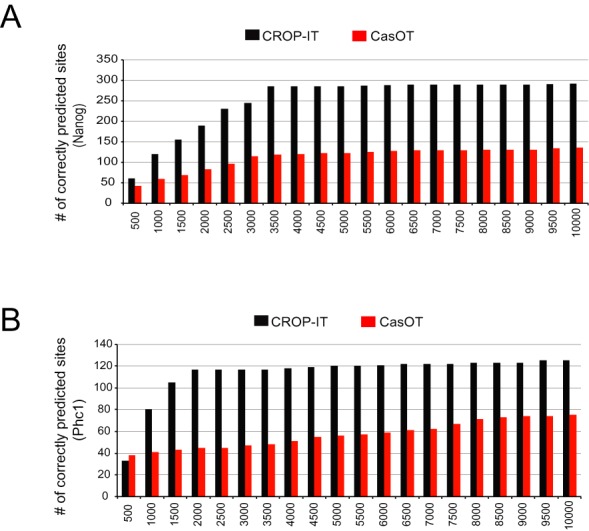
Comparative analysis of prediction performances of CROP-IT and CasOT (30) tools. Both CROP-IT and CasOT output substantial numbers of predicted sites. Thus, variable numbers of top predicted sites for each tool (X-axis) are analyzed for overlap with (**A**) *Nanog-sgRNA* and (**B**) *PhC1-sgRNA* mediated Cas9 bound ChIP-Seq sites from Wu *et al.* (22). Y-axis indicates the overlap with ChIP-Seq identified sites.

After training weights of the algorithm on experimentally identified ChIP-Seq binding sites (21), we tested it and compared the prediction power of CROP-IT to previously published five different computational tools using data from independent Cas9 binding ChIP-Seq experiment datasets from Wu *et al.* (22) and Duan *et al*. (23). We used ChIP-Seq validated whole genome Cas9 binding sites guided by three sgRNAs; *Nano-sgRNA (5957 ChIP-Seq sites)* and *Phc1-sgRNA (2948 ChIP-Seq sites)* from Wu *et al.* (22) and *EMX1 sgRNA (113 ChIP-Seq sites)* from Duan *et al.* (23). Each tool reports different numbers of predicted off-target sites. Thus to effectively compare them, we took the maximum number of off-target predicted sites from each tool and compared them with the same number of sites from CROP-IT predicted ranked list. The computationally predicted sites were then overlapped with the experimentally identified ChIP-Seq Cas9 binding sites. Notably, using these validated Cas9 binding sites as gold standard, CROP-IT performs substantially better than other tools (Figure 1B–E). To understand CROP-IT's performance compared to other tools when only limited number of top sites are considered, we performed evaluations with top 10, 100 and 200 predicted sites for Crispr.mit.edu tool (16) and CROP-IT. Figure 1F indicates that CROP-IT gives better performance when limited number of top sites are included. In cases where a substantial number of ranked off-targets are outputted, for example *CasOT tool* (30), different ranges of the top predicted sites were compared and CROP-IT predicted significantly higher number of sites (*P < 0.005; two sample test)* (Figure 2). Interestingly, we observe positive correlation between CROP-IT scores and scores from other computational tools (Supplementary Figure S1A and B) however, there seems to be no clear correlation between the computational score intensity and the limited number of experimentally validated sites (Supplementary Figure S1C). In addition to using ChIP-Seq validated Cas9 binding sites as a gold standard data, our results show that CROP-IT also outperforms other tools when commonly predicted sites from multiple tools are chosen as a gold standard (Supplementary Figure S1D).

### Incorporating chromatin state further improves CROP-IT prediction power

**Figure 3.**
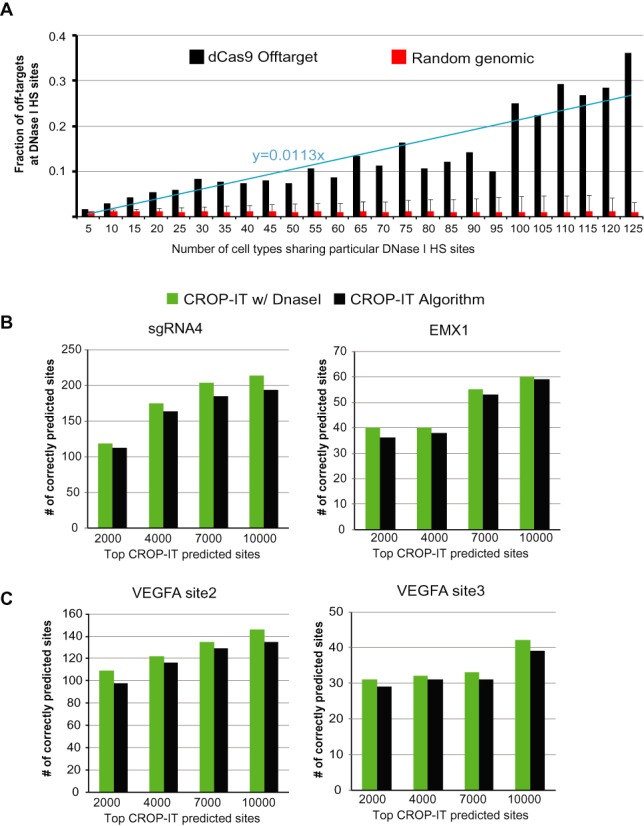
Incorporating the contribution of chromatin structure to CROPT-IT algorithm. (**A**) Percent overlap between Cas9 ChIP-Seq sites (*n* = 2600) with DNase I-seq identified DNase I hypersensitive sites (HS) from 125 different human cell types. Error bars indicate the s.d. of 1000 computational simulations for randomly selected 2600 genomic sites. X-axis display different bins of HS sites according the frequency of observation in different number of cell types. Comparison of outputs from implementing CROP-IT with DNase I chromatin state information and CROP-IT without chromatin information by overlapping (**B**) Cas9 binding sites and (**C**) Cas9 cleavage sites with predicted sites. Top 2000, 4000, 7000 and 10 000 computationally predicted sites are analyzed.

Whole-genome mapping of Cas9 binding sites showed that chromatin structure is a major component of CRISPR targeting specificity (21,22). Thus, we wanted to see if incorporating the chromatin state information into the CROP-IT scoring algorithm further improves its prediction power. To this end, we analyzed DNase I-Seq chromatin accessibility data from 125 human cell types (35). DNase I-Seq is a method that identifies ‘open chromatin’ sites at the whole genome level by mapping DNase I accessible chromatin sites (39). To better understand the contribution of chromatin structure and make this additional information applicable to wider range of different human cell types, we divided open chromatin regions (DNase I-Seq peaks) into genomic bins. These bins were then ranked according to the frequency that they appeared in different cell types. We then calculated the overlapping frequency of >2600 ChIP-Seq identified Cas9 binding sites from 12 sgRNAs in human genome (21) in these genomic bins. As a control, we simulated random regions (*n* = 2600) 1000 times and performed the same analysis. Importantly, genomic regions that are commonly open in more cell types have significantly higher probability of Cas9 binding (*P-*value = 2.82E-15) (Figure 3A).

Therefore, in order to differentially weigh genomic region according to the chromatin state information, we added an additional scoring parameter, *d*, in our scoring assignment algorithm to incorporate this information. This parameter differentially increases the score of predicted sites depending on the number of cell types displaying ‘open’ chromatin conformation for the predicted site. This additional score assignment generates a newly ranked list of predicted sequences. Since 125-cell type DNase I-Seq data is available only for the human genome, this extra feature of CROP-IT is currently available for human studies only. Re-scoring off-target sites based on chromatin state information showed consistent improvement of prediction performance for both off-target Cas9 binding sites (Figure 3B) and cleavage sites (Figure 3C). Also, the execution time per predicted sites is comparable to, if not better than, other tools (Supplementary Figure S2A). Notably, using HEK293 cell type specific DNase I Seq data, with re-trained weights, yielded comparable result to aggregate DNase I Seq data from 125 cell types (Supplementary Figure S2B).

### Evaluation of CROP-IT prediction performance for off-target cleavage sites

**Figure 4.**
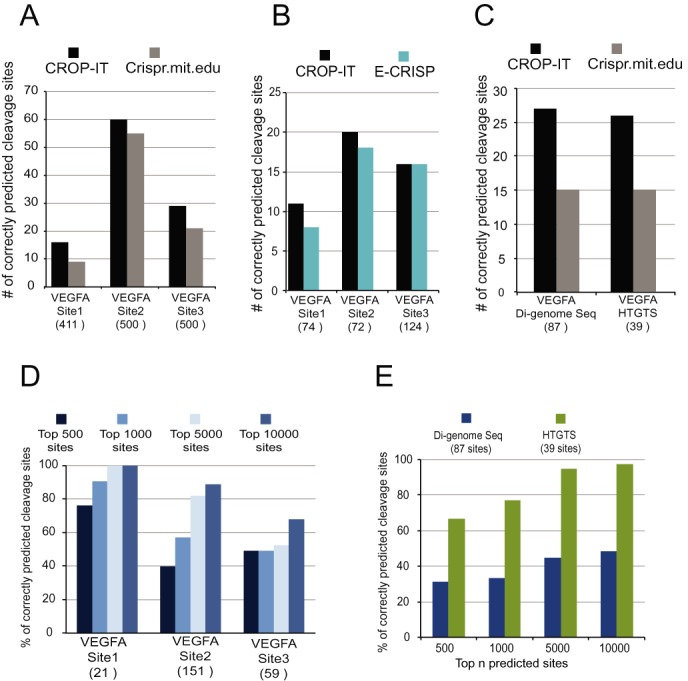
Comparative analysis of prediction performances of CROP-IT with (**A**) Crispr.mit.edu (16) and (**B**) E-CRISP (31) on GUIDE-Seq identified cleavage (24). Since each tool outputs different number of predictions, the same of sites for each tool were compared to the same number of CROP-IT predicted sites picked from the top of ranked list. Y-axis indicates the overlap with GUIDE-Seq identified Cas9 cleavage sites. (**C**) Comparative analysis of prediction performances of CROP-IT and Crispr.mit.edu (16) on cleavage sites identified through experimental HTGTS (25) and Di-genome-Seq (26) approaches for VEGFA site. Y-axis indicates the overlap with sites identified by each study. (**D**) Comparison of experimentally verified and different number of top sites predicted by CROP-IT. Y-axis indicates the percent overlap with total GUIDE-Seq identified Cas9 cleavage sites (indicated in X-axis). (**E**) Similar analysis presented in (D) was performed for HTGTS (25) and Di-genome Sequencing (26) identified Cas9 cleavage sites for VEGFA targeted guiding RNA. Y-axis indicates the percent overlap with total experimentally identified Cas9 cleavage sites and CROP-IT predicted top off-target sites (indicated in X-axis).

The above comparisons were done using catalytically inactive Cas9 ChIP-Seq binding data as gold standard. Predicting off-target Cas9 binding is important for experimental purposes where catalytically inactive Cas9 is repurposed for gene regulation, chromatin imaging and pull down. However, for genome editing purposes, it is imperative to identify off-target Cas9 cleavage sites. Thus, we evaluated CROP-IT's prediction performance of Cas9 off-target cleavage sites. To achieve this, we trained CROP-IT algorithm on the datasets recently generated by the GUIDE-Seq technology (24) which is an experimental approach used to identify whole genome level Cas9 mediated off-target cleavage sites. The training was done using the sgRNA4 cleavage sites (HEK293 Site 4) and performance evaluation was performed on data from three sites; *VEGFA* Site 1 (21 cleavage sites), *VEGFA* Site 2 (151 cleavage sites) and *VEGFA* Site 3 (59 cleavage sites). During the training we observed different weights being assigned to the three segments of the 20 bp sequence, however, following similar trend to that of off-target binding sites. We evaluated the performance of CROP-IT on three independent experimental Cas9 cleavage data sets generated by GUIDE-Seq (24), HTGTS (25) and Di-genome-Seq (26) approaches. CROP-IT performs better than state-of-the art tools Crispr.mit.edu (16) (Figure 4A) and E-CRISP (31) (Figure 4B). Notably, for the sgRNA targeting VEGFA site, recent whole genome off-target cleavage data was generated using HTGTS (25) (39 sites) and Di-genome-Seq (26) (87 sites) approaches. Comparing prediction of experimentally validated data from these two independent approaches, CROP-IT predicts substantially more number of cleavage sites (Figure 4C). Similar to previous analysis, we selected the top *N* sites from CROP-IT's output based on the number of sites output by the two tools for comparison. Unlike other tools, CROP-IT outputs significantly larger number of ranked potential off-target cleavage sites, thus, allowing users to define their own thresholds for the number of predicated sites. As expected, increasing the number of computationally predicted sites for output increases the probability of correctly predicting the actual off-target cleavage sites identified by GUIDE-Seq (24) (Figure 4D) as well as HTGTS (25) and Di-genome-Seq (26) approaches (Figure 4E). Our results presented in this study show, in principle, that integrating novel biological information into a computational algorithm significantly increases the accuracy and power of computational prediction.

## DISCUSSION

In addition to being a simple, efficient and versatile gene-editing tool, the CRISPR technology has been repurposed to achieve gene regulation, epigenetic chromatin manipulation and chromatin imaging. Thus, there is intense interest from a broad range of scientific community in the utilization of this technology. One major goal in designing the CRISPR/Cas9 experiments is to achieve the maximal targeting specificity and identify and monitor potential off-target sites. To serve this purpose, a number of computational tools have been designed to explore potential off-target sites based. In contrast to previous CRISPR design and off-target prediction tools that are based on guide RNA sequence similarity only, CROP-IT algorithm integrates crucial information obtained from experimentally validated Cas9 binding and cleavage sites from multiple independent studies (21,22,24) and chromatin state information from 125 different human cell types from diverse types and origins. CROP-IT has been devised to predict both Cas9 off-target binding sites as well as Cas9 off-target cleavage sites. Although the algorithm is same, the scores given for Cas9 binding site prediction is slightly different than the scores for Cas9 cleavage sites. Comparative analysis shows that CROP-IT outperforms existing computational tools in predicting both the actual Cas9 off-target binding sites as well as cleavage sites. Notably, CROP-IT currently uses only DNase I-Seq chromatin state information from 125 cell types. Including additional cell type specific chromatin information such as post-translational histone tail modifications and DNA methylation status might further improve the prediction power of CROP-IT for a given cell type.

It should be noted that despite substantial improvements, the ability of computational prediction tools to predict all potential CRISPR/Cas9 binding sites remains limited. Nevertheless, computational algorithms are fast and free tools that can be explored to monitor both intended and unintended Cas9 target sites. It is natural to anticipate that as we learn more about the determinants and mechanism of Cas9 targeting specificity and cleavage activity, the prediction algorithms will also improve. To accommodate such improvements in the future, CROP-IT has flexibility in its scoring scheme for including new biological information. Our study shows a proof-of-principle that taking such additional information into account in addition to sole sequence identity is a way to improve predictions and should be an area of additional research.

## AVAILABILITY

To make CROP-IT available for a broader scientific community, we made it available as a web application tool at http://www.adlilab.org/CROP-IT/homepage.html. Along with prediction of off-target binding and cleavage sites, CROP-IT also provides a feature for designing optimal sgRNA guiding sequences in which users may provide a genomic region of interest (up to 250 bp) and, in response, the tool performs a comparative analysis of potential targeting sites to predict the sgRNA guiding sequence with minimal off-target effects. CROP-IT takes ∼40 and ∼20 min to generate list of predicted binding and cleavage sites respectively, which are e-mailed to the user. The results are presented in a tab delimited file with genome coordinates of the off target site, its sequence, total number of mismatches (mismatches are represented as lower case letters), assigned score and the overlapping gene information.

## SUPPLEMENTARY DATA

Supplementary Data are available at NAR Online.

SUPPLEMENTARY DATA
