# Supplementary material for: Cas9-chromatin binding information enables more accurate CRISPR off-target prediction
Source: Nucleic Acids Res. 2015 Oct 10;43(18):e118. doi: 10.1093/nar/gkv575 (PMC4605288; doi:10.1093/nar/gkv575)
Supplement: SUPPLEMENTARY DATA [file supp_43_18_e118__index.html]

Cas9-chromatin binding information enables more accurate CRISPR off-target prediction — Cas9-chromatin binding information enables more accurate CRISPR off-target prediction — SUPPLEMENTARY DATA 

# Cas9-chromatin binding information enables more accurate CRISPR off-target prediction

## SUPPLEMENTARY DATA

- SUPPLEMENTARY DATA
