# Supplementary material for: Cas9-chromatin binding information enables more accurate CRISPR off-target prediction
Source: Nucleic Acids Res. 2015 Oct 10;43(18):e118. doi: 10.1093/nar/gkv575 (PMC4605288; doi:10.1093/nar/gkv575)
Supplement: SUPPLEMENTARY DATA [file supp_gkv575_nar-00549-met-h-2015-File006.pdf]

Supplementary Figure 1

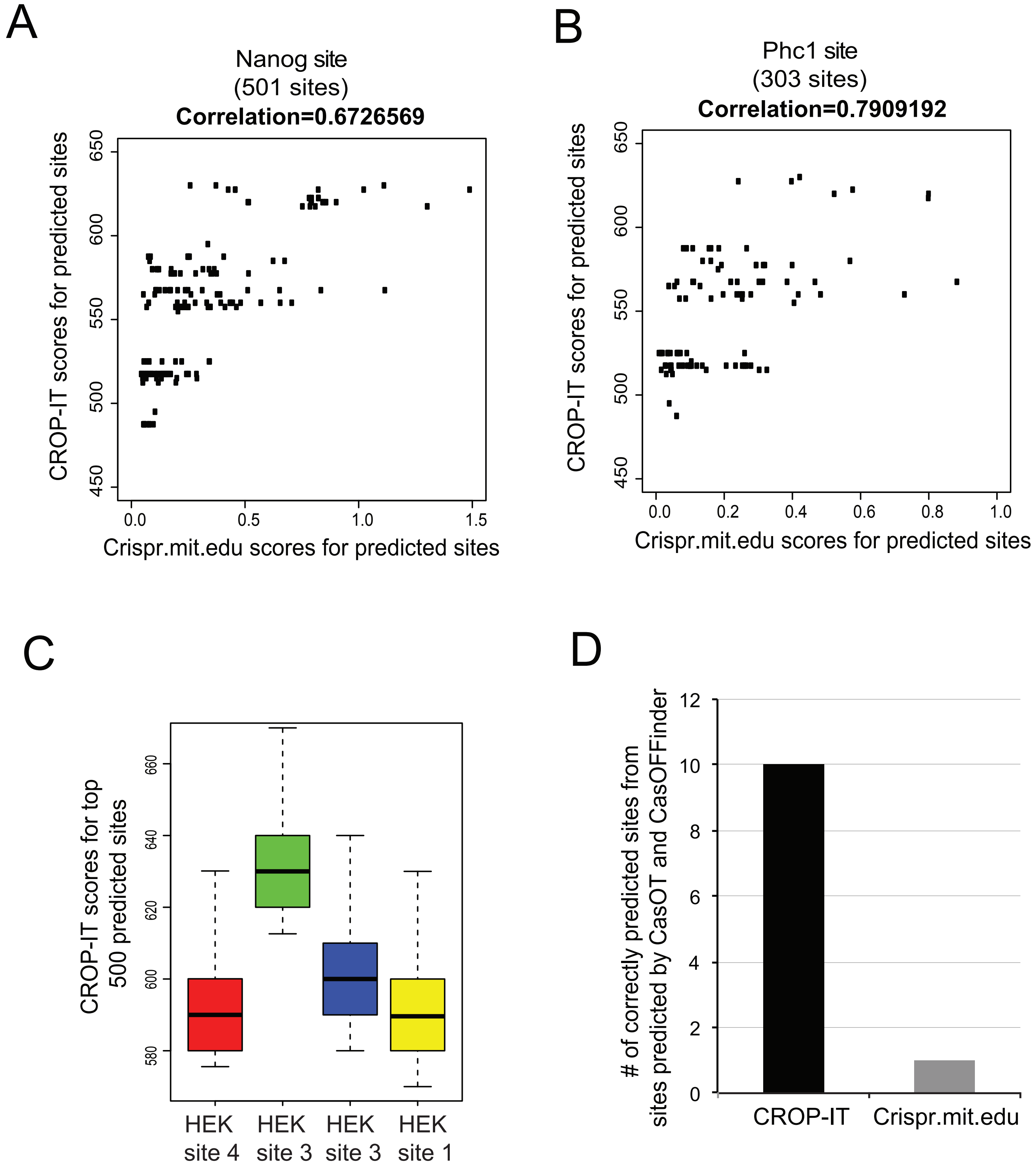

Suppl. Fig 1. Comparison of off-target scores predicted by Crispr.mit.edu (X-axis) and CROP-IT (Y-axis) for Nanog **(A)** and PHC1 sgRNAs **(B)**. **(C)** Boxplots of the scores of top 500 predcited sites by CROP-IT for the different sgRNAs used im HEK293T. **(D)** Comparison of performences of CROP-IT and Crispr.mit.edu tool on the overlapping genomic sites predicted by both CasOt tool and CasOFFinder tools.

Supplementary Figure 2

A

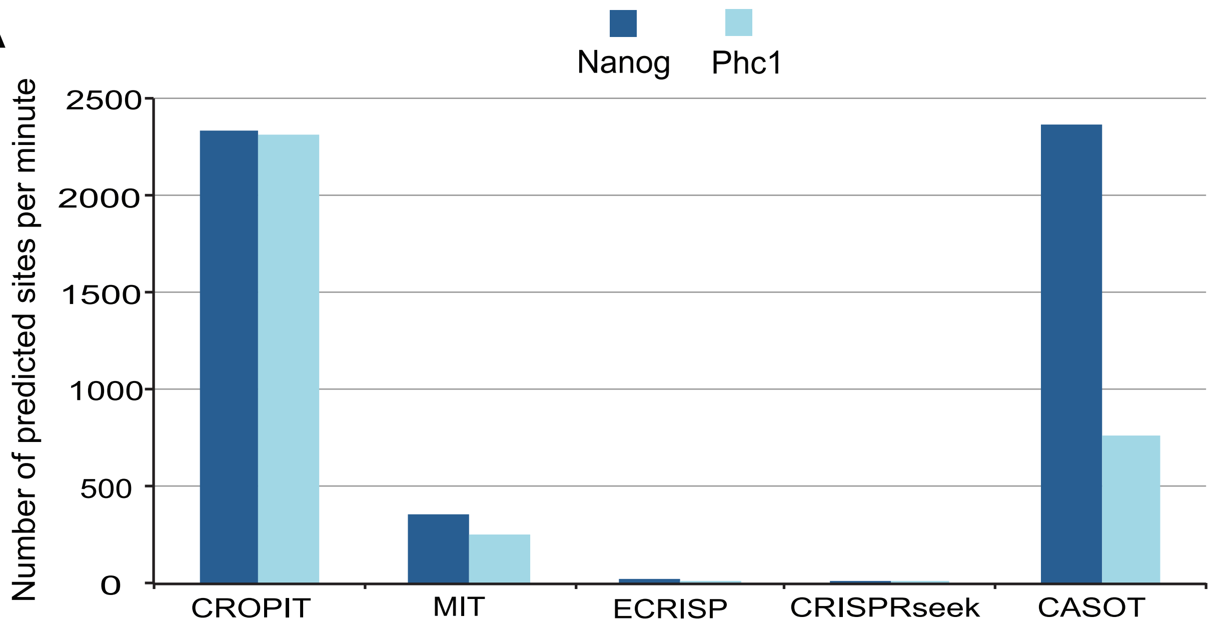

B

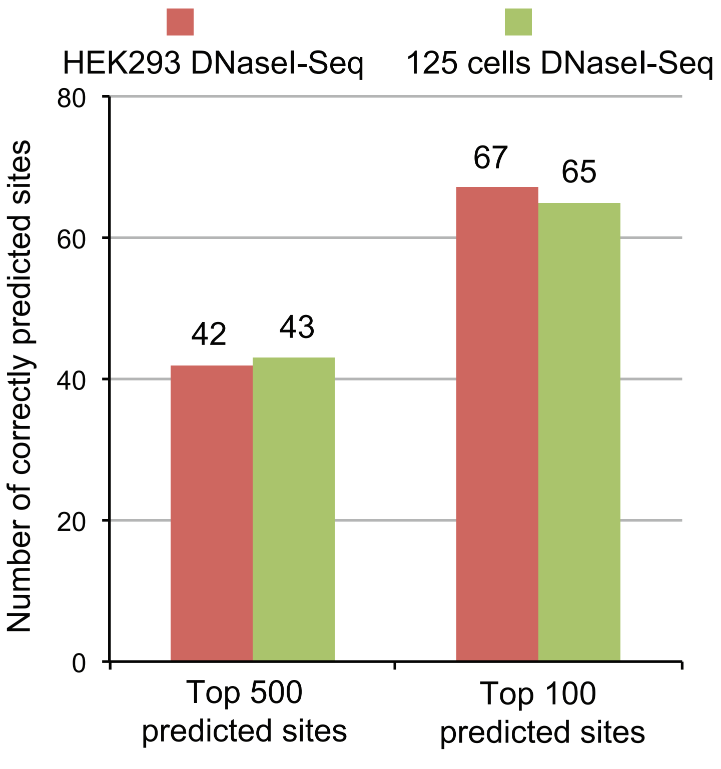

Suppl. Fig 2. (A) Comperative analysis of execution times for different computational tools. Since each tool outputs different number of predcited sites, the comparison has been done based on number of predcited sites per minute (Y-axis) **(B)** Comparison of cell type specific chromatin state data vs aggregate chromatin state of 125 cell types. Number of correctly predicted sites are shown for CROP-IT algorithm with DNAse I HS data from HEK 293 cells and aggregate DNase I HS data from 125 different human cell types.
